# Association between postpartum depression level, social support level and breastfeeding attitude and breastfeeding self-efficacy in early postpartum women

**DOI:** 10.1371/journal.pone.0249538

**Published:** 2021-04-02

**Authors:** Yeliz Mercan, Kevser Tari Selcuk

**Affiliations:** 1 Department of Health Management, School of Health Kirklareli University, Kirklareli, Turkey; 2 Department of Nurition and Dietetics, Faculty of Health Sciences, Bandirma Onyedi Eylul University, Balikesir, Turkey; International Telematic University Uninettuno, ITALY

## Abstract

**Objective:**

The study was aimed at investigating the association between postpartum women’s breastfeeding self-efficacy levels and their depression levels, social support levels, and breastfeeding attitudes in early postpartum period.

**Methods:**

The cross-sectional study was carried out in Kirklareli in Turkey. The population of the study consisted of 398 women aged 15–49 in the first 42 days of the postpartum period who presented to eight family health centers. The study data were collected face-to-face using the Personal Information Form, Breastfeeding Self-Efficacy Scale-Short Form (BSES-SF), Edinburgh Postnatal Depression Scale (EPDS), Multidimensional Scale of Perceived Social Support (MSPSS), and Breastfeeding Attitudes of the Evaluation Scale (BAES).

**Results:**

The mean age of the participants was 28.61±5.72 (Min:18, Max: 44), and the mean score they obtained from the BSES-SF was 55.13±8.39. Statistically significant differences were detected between the participants’ BSES-SF scores and age groups, employment status, perceived income level, and the number of living children (*p* < 0.05). No statistically significant differences were detected between marital status, educational status and BSES-SF scores (p > 0.05). In the multivariate regression analysis adjusted according to the sociodemographic characteristics, BAES, EPDS and MSPSS accounted for 48.3% of the BSES-SF. A negative association was found between BSES-SF scores and EPDS scores (β = −0.178, 95% CI:−0.349, −0.006), and a positive relation between the BAES scores (β = 0.194, 95% CI: 0.163, 0.226) and the MSPSS scores (β = 0.114, 95% CI: 0.037, 0.191).

**Conclusion:**

As the level of depression of women increases in the postpartum period, the level of breastfeeding self-efficacy decreases. The breastfeeding self-efficacy increases as the level of social support increases and as the attitudes that drive breastfeeding behavior change positively.

## Introduction

Breastfeeding is one of the actions effective not only on the growth and development of the baby but also on the health of the family and the community [1]. Breastfeeding best provides babies with the nutrients they need for healthy growth and development. The World Health Organization (WHO) recommends that mothers should exclusively breastfeed their children within the first six months of their lives, and then sustain breastfeeding besides supplementary foods up to the age of two years or beyond. However, approximately 40% of infants worldwide are exclusively breastfed within the first six months of their lives [2]. At the national level in Turkey, this rate was found similarly [3]. Breastfeeding rates of women are affected by many factors such as sociodemographic features, psychosocial factors, breastfeeding attitude, knowledge of breastfeeding and breastfeeding self-efficacy [4, 5].

Self-efficacy was defined as the belief in influencing the events that effect his/her life [6]. Self-efficacy, which is a dynamic process that involves cognitive, motivational, emotional, and selection processes, is also expressed as the predictor of health-related behaviors [6, 7]. Self-efficacy is one of the determinants of a range of behaviors like breastfeeding ability [8]. Breastfeeding self-efficacy reflects a mother’s confidence in breastfeeding, and affects the onset and duration of breastfeeding as a modifiable factor [8–10]. Victora et al. demonstrated that, although the main problem was the delayed onset of breastfeeding and low rate of breastfeeding in countries with low-income level, the main problem was the short duration of breastfeeding in countries with moderate and high-income levels [11]. Brockway et al. conducted a meta-analysis and found that training and support-based interventions to improve breastfeeding outcomes had positive effects on breastfeeding self-efficacy in the first 2 months after childbirth. In addition, they also found in their study that mothers in the intervention groups had a very high breastfeeding rates in the first and second months after childbirth [10]. When studies conducted at home and abroad were examined, it was found that these studies informed higher success rates in starting and continuing breastfeeding in women with high breastfeeding self-efficacy [12, 13]. The self-efficacy level, which can change with the effects of experiences in a social system, is affected by marriage relations, parenting, social status, family or professional roles, and helping spouses into the childcare [7]. When evaluated in this respect, breastfeeding self-efficacy is affected not only by performance, but also by the level of a woman’s mental health (i.e. depression, anxiety, stress) and social support [4, 13–15].

The months after the childbirth are a time of increased vulnerability to depressive mood changes, and might contribute to depressive mood changes with sudden and dramatic changes in hormones’ progesterone, estrogen, prolactin, cortisol, oxytocin, thyroid and vasopressin levels [16]. Postpartum depression refers to a non-psychotic depressive period, which starts in postpartum period, and is characterized with symptoms, such as crying, helplessness, feelings of guilt, insufficient concentration, fatigue, loss of appetite, sleep disorders, and inability feelings in dealing with the baby [17, 18]. Depressive symptomatology has a tendency to affect breastfeeding self-efficacy, which is shown with maternal confidence to breastfeed [19]. Previous studies demonstrated that there is a positive association between low breastfeeding rate and bereastfeeding duration and maternal depression, and postpartum depression poses a risk for breastfeeding self-efficacy [11, 15, 17, 20].

Perceived support defines the general perception of a person or his/her belief that people on his/her social network will provide help in a time of need. The support received, on the other hand, is complex and multidimensional because it requires that both the amount of the support given (i.e. the frequency of supporting actions, and the number of network members), and the quality of the support received are measured [17]. Zhang and Jin reported that the support received from family, friends, and other persons affected general self-efficacy and postnatal depression, and that general self-efficacy mediated the association between social support and postnatal depression [21]. Previous studies also informed that high social support levels increased the breastfeeding self-efficacy level [15, 22].

The breastfeeding attitude, which includes the belief, knowledge, and information concepts, is explained as the tendency of women to regularly display feelings, thoughts and behaviors related to a psychological object attributed to them in their breastfeeding behavior development [23]. The breastfeeding attitude of women affects the social environment, social support, successful breastfeeding, and based on this, the breastfeeding self-efficacy [5]. In a study that was conducted in South Jordan, it was reported that although the mothers had positive attitudes towards breastfeeding, approximately three-quarters of them started breastfeeding early, but only a fifth gave breastmilk for the first 6 months. Approximately one third of the mothers reported that the reason to stop breastfeeding early was starting work [24]. Balogun et al. study demonstrated that higher maternal age lowered the likelihood of having a strong intention to exclusively breastfeed an infant [14]. Women breastfeeding self‐efficacy levels, occupation, family income sufficiency were indicated as predictors of breastfeeding performance [4].

Epidemiological study demonstrated that breastfeeding has important long-term effects on health, nutrition, and development of the child and that of the mother [11]. No study was found in the region where the research was conducted on breastfeeding self-efficacy, which causes adverse health consequences and affects public health. In this region, determination of postpartum women’s breastfeeding self-efficacy levels, depression levels, social support levels and breastfeeding attitudes is important in terms of the initiatives to be planned by primary healthcare organizations to increase breastfeeding self-efficacy. In our study, we aimed to investigate the relationship between early postpartum women’s breastfeeding self-efficacy levels and their depression levels, social support levels, and breastfeeding attitudes.

## Materials and methods

### Study design and procedure

This cross-sectional study was conducted between March 2018 and August 2018 in eight Family Health Centers (FHC) in Kirklareli, which is located in Northwestern Turkey. According to the data released by the Turkish Statistical Institute in 2017, the total fertility rate was 2.07 per thousand in Turkey, and 1.54 per thousand in Kirklareli. In addition, the number of women aged 15–49 years was 26.297, the total number of births was 1.035 [25]. The minimum sampling size was found to be 280 (N = 1.035, P = 0.50, α = 0.05 and d = 0.05) in the Epi Info 7.2 program. However, due to possible refusals, withdrawals and/or losses, it was decided to include 50% more people; thus, it was aimed to reach 420 women. The inclusion criteria were being in the age range of 15–49 who reproductive-age women [26], being in the first 42 days of the postpartum period, having live birth in the last pregnancy, having a baby with no known health problem, having no known medical, obstetric or psychiatric problem, having cognitive competence to answer questions, and being a volunteer. Of the participants, 22 who did not completely respond to the questionnaire were excluded from the study. Therefore, 398 postpartum women who presented to the FHCs between the specified dates, and who were in the first 42 days of their postpartum period were reached.

### Measures

Personal Information Form, Breastfeeding Self-Efficacy Scale-Short Form, Edinburgh Postnatal Depression Scale, Multidimensional Scale of Perceived Social Support, and Breastfeeding Attitudes of the Evaluation Scale were used to collect the study data. The study was told to the women who admitted to FHCs in Kirklareli for any postpartum reason (postnatal follow-up, baby follow-up, vaccine follow-up, any disease). If they consented to take part in the study, they signed the Informed Consent Form. Those in postpartum period were asked about the date of delivery. Women whose end-of-birth dates were in the first 42 days were included in the study. The interviews were conducted in a room that was allocated for the interviews in the FHCs, and the forms were filled in by the interviewer or by the interviewee, and lasted approximately 35–40 minutes.

#### Personal information form

Prepared by the researchers, this form included questions on age, educational status, working status, perceived income level, and number of living children to determine sociodemographic characteristics.

#### Breastfeeding Self-Efficacy Scale-Short Form (BSES-SF)

The BSES-SF was firstly developed by Dennis and Faux to evaluate the self-efficacy levels of breastfeeding mothers and then the short form was created by Dennis [7]. This short form was adapted into Turkish by Alus Tokat et al. [9]. The scale was intended for determining breastfeeding mothers with high risks, evaluate breastfeeding behaviors, and cognitions to individualize confidence-building strategies, evaluate the effectiveness of various interventions, and guide future programs. Possible minimum—maximum scores that can be obtained from the scale are 14 and 70. The higher the score obtained from the scale, the higher the mother’s breastfeeding self-efficacy. The Cronbach’s alpha coefficient of the scale was found 0.86 in Alus Tokat et al.’s study, and 0.93 in the present study.

#### Breastfeeding Attitudes of the Evaluation Scale (BAES)

The BAES developed by Arslan Ozkan measures the various dimensions of attitudes (cognitive, behavioral and emotional) that affect mothers’ breastfeeding behaviors. The Cronbach’s alpha coefficient was found to be 0.86 in the scale that consisted of 46 items in 5-Point Likert style. The score that can be received from the scale ranges between 0 and 184, and high scores show positive breastfeeding attitude [27]. The Cronbach’s alpha coefficient of the scale was found to be 0.86 in Arslan Ozkan’s study, and 0.89 in the present study.

#### Edinburgh Postnatal Depression Scale (EPDS)

The EPDS, developed by Cox et al. was adapted into Turkish by Engindeniz et al. [28, 29]. The scale is a self-assessment screening scale, and is used to determine the depression risk in the postpartum period. And then Aydin et al. conducted a study to find out the validity and reliability of the Turkish version of the EPDS [30]. The scores that can be received from the 4-Point Likert style scale, which consists of 10 items, and which evaluates the psychological state in the last seven days, range between 0 and 21 points. High scores show the risk of depression or the presence of depression. The Cronbach’s alpha coefficient of the scale was found to be 0.72 in Aydin et al.’s study, and 0.77 in the present study.

#### Multidimensional Scale of Perceived Social Support (MSPSS)

The MSPSS was developed by Zimet et al. and was adapted into Turkish by Eker and Arkar [31, 32]. The scale is in 7-Point Likert style, and consists of three subscales and 12 items which subjectively measure the adequacy of social support received from the family, friends and a significant other. Lowest and highest scores are 12 and 84, respectively. The higher the score obtained from the scale, the higher the perceived social support. The Cronbach’s alpha coefficient of the scale was found to be 0.77 in Eker and Arkar’s study, and 0.75 in the present study.

### Statistical analysis

For the purpose of analyzing the study data, number (n), percentage (%), mean and standard deviation (mean ± SD) were made use of. Reliability analysis was done regarding the reliability of the scales, and the results were evaluated with Cronbach’s alpha coefficient value. The mother’s postpartum breastfeeding self-efficacy level was the dependent variable of the study, the level of social support she received, her depression level and her attitude towards breastfeeding were the independent variables. The covariates of the present study were age, education level, perceived income level, occupational status and the number of living children. According to the years in which education was received, education level was specified as illiterate (0), literate (1), primary school level (5), secondary school level (8), high school level (12) university and above (16) according to the year of education. The Student’s ***t*** Test was used to compare the mean values in two independent groups in terms of educational status, employment status, the number of living children, marital status, and the ANOVA test was used to compare the mean values in three and more independent groups in terms of age groups and perceived income levels. In the univariate analysis, variables with *p*-values < 0.10 were included in the model, and the Multivariate Linear Regression Analysis was done. The explanatory value of the models was evaluated with the Adjusted R-square (Adj. R^2^). *p*-values < 0.05 were taken as statistically significant. Analyses were done in SPSS 22.0 (SPSS Inc., Chicago, Ill., USA).

### Ethics approval

The study was approved by the Ethics Committee of the Kirklareli University Institute of Health Sciences Ethics Committee in 2018 (Date: 09.03.2018, No: P083R00) and official permission was obtained to initiate the study.

## Results

The mean age of the participants was 28.61 ± 5.72 (Min:18, Max: 44), 59.8% were in the 25–34 age group, 52.5% were graduates of secondary school or above, 98.5% were married, 30.4% had a wage-earning job, 90.7% perceived their income level as moderate. The mean number of living children was 1.58 ± 0.81 (min: 1.00, max: 6.00) (Table 1).

**Table 1:** Sociodemographic characteristics of the participants (n = 398).

*Variables*	n	%
**Age**		
≤24	97	24.4
25–34	238	59.8
≥35	63	15.8
**Education status**		
Secondary school or lower	189	47.5
High school or higher	209	52.5
**Marital status**		
Married	392	98.5
Single	6	1.5
**Employment status**		
No	277	69.6
Yes	121	30.4
**Perceived income level**		
Poor	17	4.3
Moderate	361	90.7
Good	20	5.0
**The number of living children**		
1	221	55.5
≥2	177	44.5

The mean value of participants in BSES-SF was 55.13 ± 8.39 (Min: 36, Max: 70); and 122.88 ± 23.23, 7.10 ± 4.44 and 63.82 ± 10.30, respectively in BAES, EPDS and MSPSS (Table 2).

**Table 2:** Distribution of the mean scores the participants obtained from scales.

*Scales*	N	Mean ± SD	Min.	Max.
BSES- SF	398	55.13 ± 8.39	36	70
EPDS	398	7.10 ± 4.44	2	22
MSPSS	398	63.82 ± 10.30	35	82
BAES	398	122.88 ± 23.23	65	183

BSES-SF: Breastfeeding Self-Efficacy Scale-Short Form. BAES: Breastfeeding Attitudes of the Evaluation Scale. EPDS: Edinburgh Postnatal Depression Scale. MSPSS: Multidimensional Scale of Perceived Social Support.

The comparison of breastfeeding self-efficacy level in terms of the sociodemographic characteristics is given in Table 3. A statistically significant relation was detected between the BSES-SF scores of the participants and employment status, perceived income level, and the number of living children (*p* < 0.05). No significant relations were detected between marital status, educational status and BSES-SF scores (p > 0.05) (Table 3).

**Table 3:** Distribution of the mean scores of the BSES-SF in terms of sociodemographic characteristics.

	n	Mean± SD	p
**Age**			
≤24	97	50.82±9.09	<0.001
25–34	238	56.18±7.63	
≥35	63	57.81±7.78	
**Education status**			
Secondary school or lower	189	54.90±9.23	0.602
High school or higher	209	55.34±7.58	
**Marital status**			
Married	392	55.05±8.38	0.103
Single	6	60.33±8.57	
**Employment status**			
Unemployed	277	54.55±8.49	0.036
Employed	121	56.47±8.05	
**Perceived income level**			
Poor	17	57.47±5.16	0.006
Moderate	361	55.33±8.54	
Good	20	49.60±5.64	
**The number of living children**			
1	221	54.51±9.35	<0.001
≥2	177	55.91±6.99	

In Table 4, in the multivariate regression analysis adjusted according to the sociodemographic characteristics, BAES, EPDS and MSPSS accounted for 48.3% of the BSES-SF. Although statistically significant negative associations were detected between BSES-SF and EPDS scores (*β* = −0.178, 95% CI:−0.349, −0.006), there was a statistically significant positive association between the BAES scores (*β* = 0.194, 95% CI: 0.163, 0.226) and the MSPSS scores (*β* = 0.114, 95% CI: 0.037, 0.191).

**Table 4:** Multivariate linear regression analysis with BSES-SF averages of the participants.

	Unadjusted	†Adjusted
	β (95% CI)	β (95% CI)
MSPSS	0.356 (0.284, 0.429)***	0.114 (0.037, 0.191)**
EPDS	−0.693 (−0.866, −0.519)***	−0.178 (−0.349, −0.006)*
BAES	0.241 (0.214, 0.267)***	0.194 (0.163, 0.226)***

*p < 0.05

**p < 0.01

***p < 0.001. Adjusted R^2^: 0.483, F = 47.312***.

BSES-SF: Breastfeeding Self-Efficacy Scale-Short Form. BAES: Breastfeeding Attitudes of the Evaluation Scale. EPDS: Edinburgh Postnatal Depression Scale. MSPSS: Multidimensional Scale of Perceived Social Support.

## Discussion

The present study aimed at investigating the associations between postpartum women’s breastfeeding self-efficacy levels and their depression levels, social support levels and breastfeeding attitudes to provide an opportunity to determine self-efficacy levels of postpartum women living in the northwest of Turkey and to reveal related factors. Evidence has demonstrated that breastfeeding self-efficacy is an important determinant of the current intention to breastfeed and of the duration of breastfeeding [5, 8, 13].

While mothers with low breastfeeding self-efficacy had difficulty in starting breastfeeding, or stopped breastfeeding early, mothers with a moderate or high breastfeeding self-efficacy level continued breastfeeding for longer periods of time [13, 33, 34]. In the current study, the mean BSES-SF score (55.13 ± 8.39) for women in the postpartum period living in Kirklareli was similar to the women of the postpartum period living in British Columbia (55.88 ± 10.85) and Izmir Turkey (56.19 ± 8.62); lower than women in postpartum period living in Brazil (63.51 ± 6.25) and in Sakarya in Turkey (61.02 ± 8.44); higher than women in postpartum period living in China (47.28 ± 10.56) [7, 12, 19, 34, 35]. Among the studies whose findings were given here, the study of Zubaran & Foresti evaluated breastfeeding self-efficacy until 12^th^ week. In the study of Çınar et al., the mean age of the women was lower than our study group, and Denis conducted a longitudinal study until 8^th^ week [7, 19, 35]. These differences between the aforementioned studies are thought to stem from the differences between the designs of the studies and between the durations of postpartum periods included in them.

In our study, age, working status, perceived income levels and number of living children were found to be among the sociodemographic factors that affected breastfeeding self-sufficiency. Ngo et al. showed that mothers’ breastfeeding self-efficacy and profession were related [15], Pakseresht et al. showed that employment status was related [33], Corby et al. showed that children’s number, income levels, education and marital status were related [36], Chaput et al. showed that low socioeconomic status, parity, mother’s educational status, and marital status were related [37]. Unlike these studies, which support our results, our study found that there were no relations between educational status and marital status. Breastfeeding self-efficacy, which is affected by previous breastfeeding experiences and social support levels [15, 20] might have reduced the difference in educational status, or the fact that almost all of our group (98.5%) consisted of married women might have affected our results. Alghamdi et al., who conducted a similar finding with our study, showed that age was determinant, but could not determine any relations between educational status, marital status, and breastfeeding efficacy [38]. In addition, the mother’s psychological status (e.g. depression, anxiety, stress) in the postpartum period the source of social support (husband, mother, father, caregiver etc.) and breastfeeding attitude are stated among the determinants of breastfeeding self-efficacy [22, 35].

Hormonal processes protecting mothers against postpartum depression by attenuating cortisol response to stress can be enhanced by breastfeeding [39]. However, in the literature, low socioeconomic status, low level of social support, pregnancy-related negative experiences, delivery and postpartum period, and low level of self-confidence have been shown among the determinants of postpartum depression level, which affects the mother’s breastfeeding performance and breastfeeding self-efficacy [37]. While in Haga et al.’s study, breastfeeding self-efficacy was reported to predict changes in postpartum depressive symptoms, in Zhang and Jin’s study, self-efficacy was demonstrated to partially mediate the association between social support and postpartum depression [20, 21]. In the present study, a negative association was determined between the participating mothers’ breastfeeding self-efficacy levels and their postpartum depression levels. This result is consistent with the results of studies in the literature [15, 19, 37].

The level of social support a woman receives from her spouse, mother or other family members or nurses/ midwives during the breastfeeding process affects her breastfeeding self-efficacy positively [15, 19]. Consistent with the literature, in the present study, a positive association was detected between breastfeeding self-efficacy level and social support level [5, 36]. Professional support received from health professionals also plays an important role in increasing breastfeeding self-efficacy. Evidence-based studies showed that interventions by health professionals to mothers and/or fathers significantly increased breastfeeding self-efficacy [34, 40]. Brockway et al. reported that in interventions based on training or support, each 1-point increase in breastfeeding self-efficacy increased breastfeeding rates by 10% in the intervention group, which suggests that breastfeeding self-efficacy is a modifiable factor practitioners can aim to improve breastfeeding rates in mothers [10].

Attitude is defined as spontaneous and unconscious beliefs about a particular behavior [41]. A woman’s breastfeeding attitude is associated with many factors such as maternal motivation, determination, previous experiences, psychological adjustment, and attitude towards pregnancy [21, 42]. Balogun et al. showed that advanced maternal age and an unintended pregnancy negatively affected mothers’ breastfeeding intention, and that prenatal exclusive breastfeeding intention was a strong predictor of exclusive breastfeeding [14]. In order to maintain continuous breastfeeding, which provides a strong bond between the mother and the baby, the woman must have a positive breastfeeding attitude and breastfeeding intention [5, 42]. The breastfeeding attitude is a dynamic process that is influenced not only by the personal characteristics of the woman but also by several cultural factors that exist in her family, social environment or geographical region in which she lives [23]. As reported in several studies, not only maternal motivation and maternal behaviors but also the husbands’ and caregivers’ attitudes are among the factors affecting mothers’ self-efficacy levels [19, 40, 42]. In fact, in the present study, in parallel with the literature, it was found that a woman’s breastfeeding attitude was related to her breastfeeding self-efficacy, and that the more positive the attitude displayed by the mother towards breastfeeding, the higher her breastfeeding self-efficacy.

The fact that this study was conducted with women in early postpartum period, which included the first 42 days, provides data on breastfeeding efficacy in this period. This is the strength of the study. However, this study also had some limitations. The first limitation was that the cause-and-effect relation could not be revealed completely because of the cross-sectional design of the study. For this reason, the results of this study should be interpreted with caution. Secondly, since the study was conducted with women in the first 42 days of their postpartum period presenting to eight FHCs, the results could be generalized to this group. Another limitation was the evaluation of self-efficacy levels in a self-reported manner. Another limitation was that breastfeeding self-efficacy was not investigated in adolescent mothers.

## Conclusions

In the present study, it was detected that the breastfeeding self-efficacy of postpartum women increased as their depression level decreased, social support level increased and breastfeeding attitudes improved. In this respect, it is recommended that healthcare personnel should assess women’s breastfeeding self-efficacy and breastfeeding capacity routinely to detect breastfeeding problems earlier. Not only in postpartum period, but also as of prenatal period, women should be regularly screened in FHCs for depression during the follow-ups, and referred to a secondary healthcare center for psychiatric support whenever necessary. Also, women should be supported with physical activities, participation to the society, and educational counseling programs, which improve health, to eliminate inadequate self-efficacy levels, which might occur as a result of negative experiences during pregnancy. Healthcare personnel should seek out social support sources for women, and ensure that women utilize these sources effectively. In addition, healthcare personnel should also plan and implement training and counseling programs aiming to encourage women to develop a positive attitude towards breastfeeding. Spouses or families should participate in these programs by prioritizing sociodemographically disadvantaged groups, and efforts should be made to support breastfeeding in a positive way.

## Supporting information

S1 Fig (PNG)

S1 File Oral presentation English translated. (DOCX)
