# Supplementary figures and images for: Association between postpartum depression level, social support level and breastfeeding attitude and breastfeeding self-efficacy in early postpartum women
Source: PLoS One. 2021 Apr 2;16(4):e0249538. doi: 10.1371/journal.pone.0249538 (PMC8018654; doi:10.1371/journal.pone.0249538)

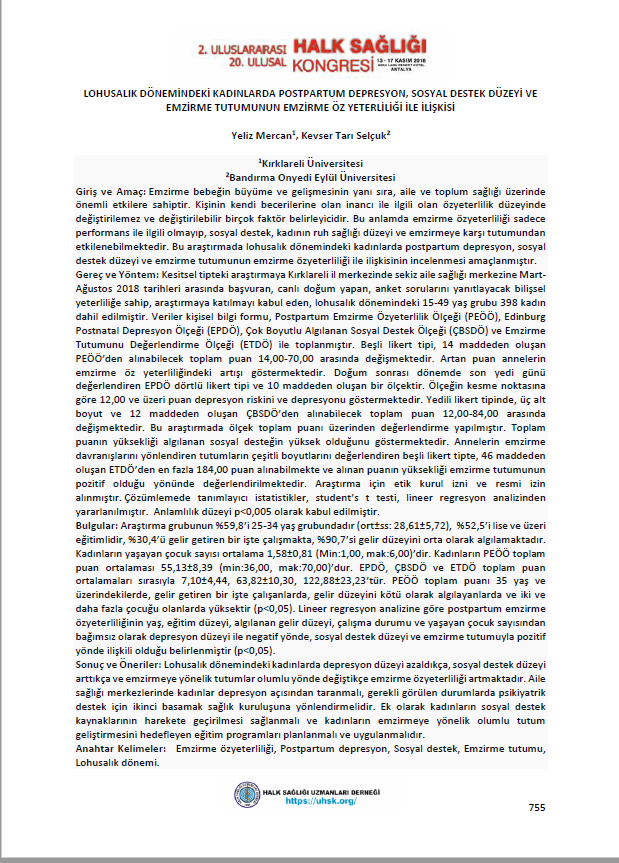

Supplement: S1 Fig — (PNG) [file pone.0249538.s001.png]
