# Supplementary material for: Association between postpartum depression level, social support level and breastfeeding attitude and breastfeeding self-efficacy in early postpartum women
Source: PLoS One. 2021 Apr 2;16(4):e0249538. doi: 10.1371/journal.pone.0249538 (PMC8018654; doi:10.1371/journal.pone.0249538)
Supplement: S1 File — (DOCX) [file pone.0249538.s002.docx]

**Oral Presentation English Translated**

**Association between postpartum depression level, social support level and breastfeeding attitude and breastfeeding self-efficacy in postpartum women**

**Background and aim:** Breastfeeding is one of the actions effective not only on the growth and development of the baby but also on the health of the family and the community. Fixed factors and variable factors are the predictors of a woman’s level of self-efficacy and belief in her own skills. In this sense, breastfeeding self-efficacy not only is related to performance, but also is affected by the woman's mental health social support level and attitude towards breastfeeding. In this study aims to investigate the relationship between early postpartum women’s breastfeeding self-efficacy levels and their depression levels, social support levels, and breastfeeding attitudes.

**Materials and Methods:** The cross-sectional study included 398 puerperal women aged 15-49 who applied to eight family health centers (FHCs) in Kirklareli city center between March and August 2018, had a live birth, had cognitive competence to answer the questionnaire questions, agreed to participate in the study . The study data were collected using the Personal Information Form, Breastfeeding Self-Efficacy Scale-Short Form (BSES-SF), Edinburgh Postnatal Depression Scale (EPDS), Multidimensional Scale of Perceived Social Support (MSPSS), and Breastfeeding Attitudes of the Evaluation Scale (BAES). Five-point Likert type, consisting of 14 items the total score that can be obtained from BSES-SF varies between 14.00-70.00. Increasing points mother's shows the increase in breastfeeding self-efficacy. The EPDS evaluating the last seven days in the postpartum period is a four-point likert-type scale consisting of 10 items. According to the cut-off point of the scale a score of 12.00 and above indicates the risk of depression and depression. Seven-point Likert type, three sub dimension and consisting of 12 items the total score that can be obtained from MSPSS varies between 12.00-84.00. In this study, evaluation was made on the scale total score. The higher the total score, the higher the perceived social support. Five-point Likert type, consisting of 46 items the total score that can be obtained from BAES varies between 0-184.00. The higher the score is the more positive the mother’s breastfeeding attitude towards breastfeeding is. Ethics committee approval and official permission for research was obtained. In the statistical analysis, descriptive statics, student’s t test, linear regression analysis were used. p < 0.05 was used to assess for significance.

**Results:** Of the participants in the present study, 59.8% were in the 25-34 age group (mean ± SD: 28.61 ± 5.72), 52.5% had high school and higher education, 30.4% had a wage-earning job, 90.7% perceived their income level as moderate. The mean number of living children was 1.58 ± 0.81 (Min: 1.00, max: 6.00). The mean score the participants obtained from the overall BSES-SF was 55.13 ± 8.39 (min: 36.00 max: 70.00). The mean scores they obtained from the EPDS, MSPSS and BAES were 7.10 ± 4.44, 63.82 ± 10.30 and 122.88 ± 23.23, respectively. Of the participants, those who were ≥35 years old, who had a wage-earning job, who perceived their income level as bad and who had two or more children obtained higher scores from the BSES-SF (p <0.05). According to linear regression analysis, while postpartum breastfeeding self-efficacy was negatively correlated with variables such as age, education level, perceived income level, employment status and the depression level independently of the number of living children, it was positively correlated with the social support level and breastfeeding attitude (p <0.05).

**Conclusion and Recommendations**: The breastfeeding self-efficacy of puerperal women increased as their depression levels decreased, social support levels increased and breastfeeding attitudes changed for the better. In FHCs, women should be screened for depression, and if necessary, referrals to secondary health care institutions for psychiatric support. In addition, women's social support resources should be activated and training programs aimed at developing a positive attitude towards breastfeeding should be planned and implemented.

**Key words**: Breastfeeding self-efficacy, Postpartum depression, Social support, Breastfeeding attitude, Postpartum period.
